# Eczema

**Published:** 1890-10

**Authors:** Wm. Steinrauf

**Affiliations:** St. Charles, Mo.


### ECZEMA.

Wm. Steinrauf, M. D., St. Charles, Mo.

It was in the fall of 1881 that a young man left the State of Missouri and moved to a country town in Illinois. His health before this time was all that could be desired. The following summer he had a peculiar breaking-out on the face of small pimples filled with an offensive-looking and offensive-smelling matter. A slight itching sensation accompanied the pimples. The trouble began sometime in July and lasted till sometime in November. The following summer eczema appeared on the left side of the face that was very annoying, and by scratching was carried to the right side of the face, the neck, and the scrotum. He was treated by all known systems, but all to no purpose. No remedy or external application received at the hands of the best physicians of all schools had any effect in either relieving or curing the patient. Chloroform was generally always inhaled at night in order to procure sleep. Nothing did any good. In the fall all would be well, although after shaving the face would be intensely sensitive for a few hours. Thus the patient lingered on from year to year, a living monument of the physician's inability and ignorance. The scratching indulged in was something frightful to behold. Hot water freely applied to the face was about the only thing that would, for the time being at least, assuage the trouble. The only medicated external application to do any good was recommended by a lady friend. It consisted in steeping the green leaves of the deadly night-shade in hot milk and freely bathing the face in it. I found on examining this weed that it was not the deadly night-shade properly speaking, but only a species of it, the Solanum nigrum. This grows wild all over our Western States in fence corners, dark and moist places.

The face at times would swell up enormously, giving the man an awful appearance. In the intense agony ice was applied one evening and did, indeed, soothe the sufferer wonderfully, but the next morning matters were worse than ever. The eyes were closed and the itching intensified. The patient, after thus suffering for over seven years, talked of a change of climate.

For several years he had noticed that when going to the timber, coming in contact with weeds of any kind, climbing a tree, or going up in the hay-loft his eczema would get much worse. But as all these conditions occur more or less in every case of eczema, the remedy could not be found in this direction. All the known remedies for eczema were tried in his case, the symptomatology compared, and medicines, high and low, given, but the result was *nil.*

But there is a saying that shortly before day the night is blackest, and when the trouble is greatest help is nearest. So it happened here.

The young man had noticed that by remaining home nights there would be less troublesome itching, and he had further noticed—and this was considered an item of importance—that in going to the barn about ten o'clock at night to see if all was well with the stock he would have to pass a large bunch of deadly night-shade, when the itching would be very much worse.

Seeing that the patient always got relief by steeping the green leaves of the deadly night-shade in milk and freely bathing the face in it, might it not be possible that the dynamized drug of this place could cure the patient? Although there is no proving of the Sol-nig. as far as I know, I was willing to try and see the result. I wrote to Dr. Swan for the DMM of the Sol-nigrum and the man took one dose every four hours till four doses had been taken, with the result that within one week the eruption and the itching were gone and the sufferer of eight years was cured. Two years have now elapsed and there is no recurrence of the trouble.

